# Tumor microenvironment-responsive BSA nanocarriers for combined chemo/chemodynamic cancer therapy

**DOI:** 10.1186/s12951-022-01442-5

**Published:** 2022-05-12

**Authors:** Ruiyi Zhang, Teng Liu, Wanzhen Li, Zhiyuan Ma, Pei Pei, Weiwei Zhang, Kai Yang, Yugui Tao

**Affiliations:** 1 School of Biological and Food Engineering, Anhui Polytechnic University, Wuhu, 241000 Anhui China; 2 State Key Laboratory of Radiation Medicine and Protection, School of Radiation Medicine and Protection & School for Radiological and Interdisciplinary Sciences (RAD-X), Collaborative Innovation Center of Radiation Medicine of Jiangsu Higher Education Institutions, Soochow University, Suzhou, 215123 Jiangsu China

**Keywords:** Tumor microenvironment, Chemodynamic therapy, Chemotherapy, Bovine serum albumin, Nanoscale coordination polymers

## Abstract

Tumor microenvironment (TME), characterized by high glutathione (GSH), high hydrogen peroxide (H_2_O_2_) and acidic pH levels, is favorable for the growth, invasion and metastasis of cancer cells. Taking advantage of the specific characteristics of tumors, TME-responsive GCBD NPs are designed to deliver nanoscale coordination polymers (NCPs, GA-Cu) and chemotherapy drugs (doxorubicin, DOX) based on bovine serum albumin (BSA) nanocarriers into cancer cells for combined chemodynamic therapy (CDT) and chemotherapy. In an acidic environment, GCBD NPs could release approximately 90% copper ions, which can not only consume overexpressed GSH to modulate the TME but can also react with endogenous H_2_O_2_ in a Fenton-like reaction to achieve the CDT effect. Meanwhile, the released DOX could enter the nucleus of tumor cells and affect their proliferation to achieve efficient chemotherapy. Both in vitro and in vivo experiments showed that GCBD NPs had good biosafety and could effectively inhibit the growth of cancer cells. GCBD NPs are promising as a biocompatible nanoplatform to exploit TME characteristics for combined chemo and chemodynamic therapy, providing a novel strategy to eradicate tumors with high efficiency and specificity.

**Graphical Abstract:**

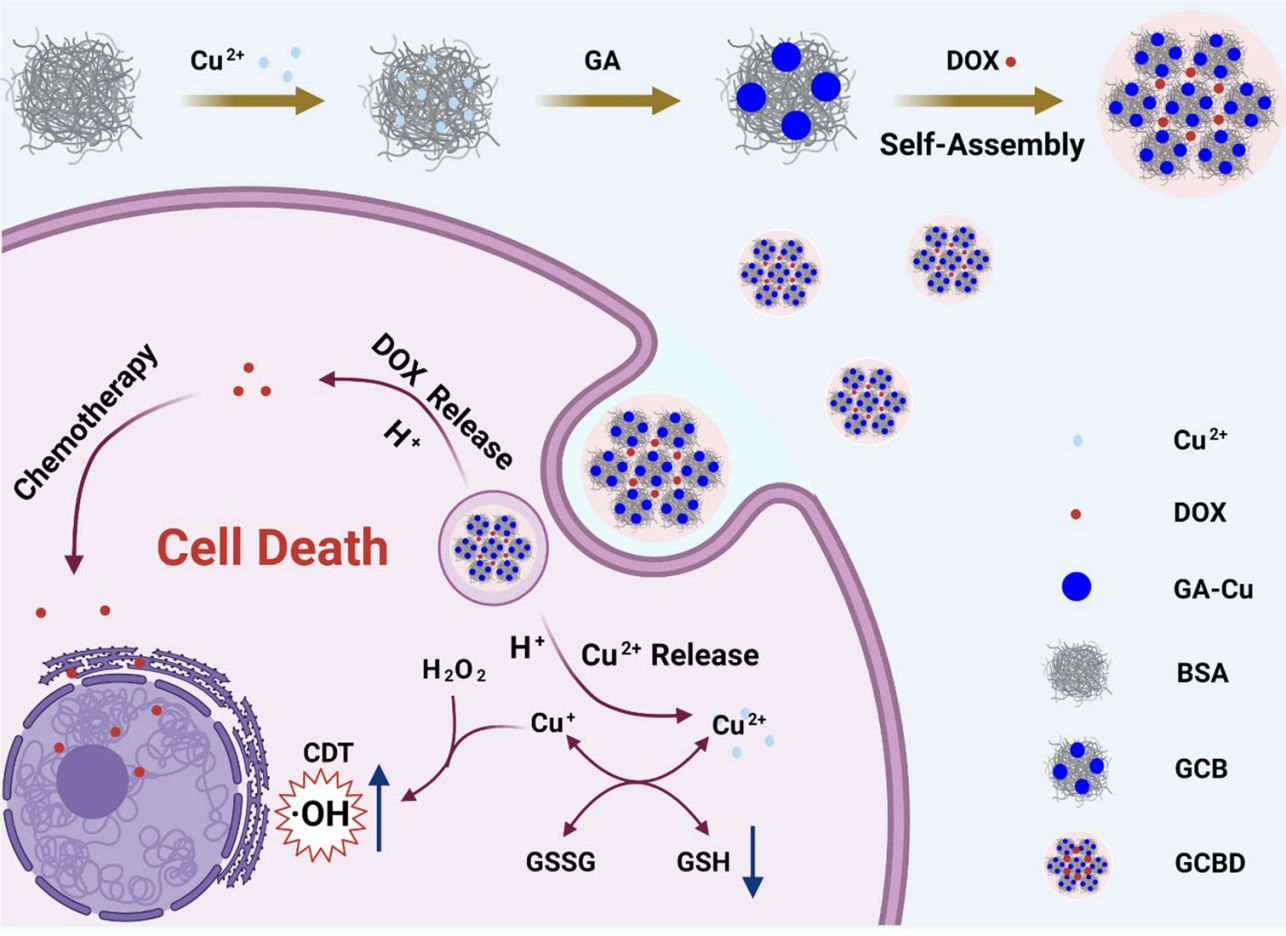

**Supplementary information:**

The online version contains supplementary material available at 10.1186/s12951-022-01442-5.

## Introduction

The tumor microenvironment (TME) is an acidic environment that contains various cell types (such as tumor cells, immune and inflammatory cells and tumor-related fibroblasts, etc.) and extracellular matrix components with high glutathione (GSH), high hydrogen peroxide (H_2_O_2_) and low oxygen (O_2_) contents [1, 2]. The interaction between tumor cells and the complicated TME is responsible for drug resistance, limited therapeutic efficacy and invasion and metastasis after various treatments [3, 4]. It has been recognized that the modulation of the TME by promoting ROS (reactive oxygen species) and/or reducing ROS scavenging agents (e.g., GSH) could effectively ablate cancer cells by elevating intracellular oxidative stress [5–7]. Chemodynamic therapy (CDT) based on the Fenton reaction, which catalyses hydrogen dioxide (H_2_O_2_) into highly toxic hydroxyl radicals (∙OH) by iron ions, has emerged as a novel therapeutic approach for cancer through oxidative damage of lipids, proteins and DNA [8]. Recently, copper-based Fenton-like reactions with higher reaction rates in a broader pH range showed more possibilities for highly efficient CDT [9–13]. However, copper is a trace element in the body incapable of inducing sufficient therapeutic efficacy alone, which would induce toxicity to normal cells at high concentrations [14]. In order to enhance the anticancer efficacy and meanwhile compromise the side effects, it is an urgent need to integrate CDT with other therapeutic modalities.

Chemotherapy using toxic drugs has been one of the most pivotal methods to treat cancer over the past decades, while the clinical outcome is severely hindered by unsatisfactory antitumor efficacy and various side effects [15]. Among them, the anthracycline antibiotic doxorubicin (DOX) as an FDA-approved chemotherapeutic agent has been universally adopted to treat various cancers through intercalation between base pairs in the DNA helix to prevent DNA replication [16–18]. Moreover, it has been reported that DOX could activate nicotinamide adenine dinucleotide phosphate oxidases (NOXs) to catalyze nicotinamide adenine dinucleotide phosphate (NADPH) and O_2_ to produce NADP+ and superoxide [19]. Subsequently, in the presence of superoxide dismutase (SOD), superoxide would be disproportionate into oxygen and H_2_O_2_ [20, 21], which could further fuel CDT. Hence, it would be of great promise for efficient cancer treatment by developing a biocompatible nanocarrier to specifically deliver and release DOX and copper ions into the tumor site for combined chemo and chemodynamic therapy. Bovine serum albumin (BSA) is a biocompatible globulin in bovine serum, which has wide applications in nanomedicine [22–26]. The hydrophobic region on the protein is feasible for loading hydrophobic chemodrug such as DOX [27–30], while its abundant amino and cysteine groups tend to attach metal ions such as copper ions [31, 32]. However, the metal ions attached to albumin are prone to uncontrolled release without specificity, leading to unsatisfactory copper dosages for CDT and latent toxicity issues [33, 34]. Gallic acid (GA) is a polyphenol derived from tea that has been widely used in the pharmaceutical industry due to its economical and accessible extraction procedure [35]. Several nanoscale coordination polymers (NCPs) have been synthesized through the chelation reaction between metal ions and the carboxyl and phenolic hydroxyl groups of GA [36, 37].

Herein, we synthesize a novel nanomedicine (GA-Cu@BSA-DOX, GCBD NPs) based on BSA nanocarriers for combined chemo and chemodynamic cancer therapy. GA-Cu nanodots (GC NDs) are formed by the in-situ coordination of GA and copper ions absorbed in BSA, which subsequently gather together by hydrophobic interactions upon the addition of doxorubicin (DOX), forming GCBD NPs with larger size. Under acidic conditions, the GCBD NPs would simultaneously release copper ions (Cu^2+^) and DOX. The released Cu^2+^ could consume GSH and produce Cu^+^, which subsequently induces a Fenton-like reaction by catalysing a high content of H_2_O_2_ in the TME to generate highly cytotoxic hydroxyl radicals (·OH) for cancer cell death. Meanwhile, the enhanced drug delivery and specific DOX release based on the BSA nanocarrier would further kill tumor cells by inhibiting their DNA replication and produce more H_2_O_2_ to fuel CDT. The residual chemical groups in GCBD NPs could be stably labeled with radionuclide ^125^I to evaluate the blood circulation and biodistribution behavior in mice [38–40]. Taking advantage of the efficient tumoral accumulation and specific release of DOX and Cu^2+^, GCBD NPs would strongly inhibit tumor growth by a synergistic effect. In this study, we designed a biocompatible nanoplatform to exploit TME characteristics for combined chemo and chemodynamic therapy, providing a novel strategy to eradicate tumors with high efficiency and specificity.

## Materials and methods

### Materials

Bovine serum albumin (BSA) and 2′,7′-dichlorofluorescin diacetate (DCFH-DA) were purchased from Sigma-Aldrich Co. Polyvinylpyrrolidone (PVP) was purchased from Alfa Aesar (China). Gallic acid (GA) and disodium terephthalate (TA) were purchased from Aladdin (Shanghai). Copper (II) chloride was purchased from Siuopharm Chemical Reagent Co. The GSH/GSSG assay kit, live/dead assay kit, calcein-AM, propidium iodide (PI) and hydrogen peroxide assay kit were obtained from Beyotime Biotechnology. CCK-8 was obtained from APExBIO. HCl·DOX was obtained from Meilunbio.

### Synthesis of nanomaterials

#### PVP stabilized GA-Cu nanodots (GC NDs)

60 mg PVP was dissolved in 8 mL deionized (DI) water under stirring, followed by dropwise addition of 1 mL CuCl_2_·2H_2_O (10 mg/mL) and 1 mL GA (10 mg/mL) for reaction overnight. The as-prepared GC NDs were then purified by centrifugation at 3500 rpm for 10 min using ultrafiltration Millipore tubes (10 kDa) and washed three times with DI water.

#### GA-Cu@BSA nanodots (GCB NDs)

70 mg BSA was dissolved in 8 mL DI water under gentle stirring for 5 min. Then, 1 mL CuCl_2_·2H_2_O (10 mg/mL) was added dropwise with constant stirring for 30 min. After that, 1 mL 10 mg/mL GA was added to the bottle with vigorous stirring for another 3 h. After the reaction, the agglomerates were removed by centrifugation (4000 rpm). Unreacted impurities were removed with an ultra-filtration (100 kDa, 3500 rpm) washed twice with DI water and then stored in a refrigerator at 4 °C for further application.

#### GA-Cu@BSA-DOX nanoparticles (GCBD NPs)

10 mg DOX dissolved in 200 µL DMSO was added dropwise into 1 mL 10 mg/mL GCB solution with vigorous stirring for 12 h. After the reaction, the free DOX and DMSO were removed by ultra-filtration (100 kDa, 3500 rpm), washed with DI water for three times (until the UV–Vis absorption peak of DOX in the liquid below was less than 0.2) and then stored in a refrigerator at 4 °C for further use.

### Characterization of nanomaterials

The morphology and size of GA NDs, GCB NDs and GCBD NPs were characterized by TEM (Tecnai G2 spirit Bio Twin). Their physiological size and zeta-potential were tested by DLS (Zetasizer Nano ZS90). The absorbance spectra were tested by UV–Vis spectroscopy (GENESYS 10 S Spectrophotometer). Fourier transform infrared (FT-IR) spectra were recorded by conventional Fourier infrared spectroscopy (NICOLET iS 50). XPS spectra were tested by Thermo Scientific K-Alpha. In order to test the physiological stability, GCB NDs and GCBD NPs were dissolved in PBS, water and DMEM/high glucose culture medium, respectively (n = 3). Every other day, the solutions were photographed, and the size and PDI value were measured by DLS for 7 days.

### Drug loading and release behavior

#### Drug loading capacity of GCB NDs

In order to test the ability of GCB NDs to load drugs, 10 mg GCB NDs was dissolved in 1 mL DI water. Then, DOX solutions with different concentrations (20 mg/mL, 10 mg/mL, 5 mg/mL, 2.5 mg/mL and 1.25 mg/mL) were added to GCB ND solutions for reaction overnight. After the reaction, the free DOX and DMSO were removed by ultrafiltration (100 kDa, 3500 rpm).

#### Release of DOX

In order to test the DOX release behavior, 10 mg GCBD NPs was dissolved into 2 mL DI water in a dialysis bag, which was put into bottles containing 15 mL PBS (pH = 5.6 or 7.4) for stirring (3 parallel samples were set in each group). At 30 min, 1 h, 2 h, 4 h, 6 h, 12 and 24 h after stirring, 1 mL of the solutions outside the dialysis bag was removed to record the absorbance intensity at 500 nm by UV–Vis and then put back to the system.

#### Release of copper ions

The release of Cu^2+^ from GCBD NPs was detected by Copper Determination Kit (Beyotime Biotechnology). Generally, 10 mg GCBD NPs was dissolved in 2 mL DI water, which was then put into dialysis tube with PBS (pH 5.6 and pH 7.4) (3 parallel samples were set in each group) outside, 10 µL was taken at intervals of 5 min, 30 min, 1 h, 2 h, 6 h, 12 and 24 h. The same volume of PBS solution was then added to the reaction system.

### Labeling Cy5.5 and ^125^I onto GCBD NPs

#### Cy5.5 labeling

10 mg GCB NDs and GCBD NPs were dissolved in 1 mL of DI water. Subsequently, 10 µL aminated Cy 5.5 was added into the solution and stirred for 2 h under dark conditions. Excess Cy5.5 was removed with 10 kDa (for Cy5.5-GCB) and 100 kDa (for Cy5.5-GCBD) ultra-filtration tube and the products were stored at 4 °C in a dark environment for further use.

#### ^125^I labeling

2.21 mg 1,3,4,6-Tetrachloro-3α,6α-diphenyl-glycouril (Idongen) was dissolved into 600 µL chloroform with chloroform and later blow-dried with nitrogen. Then, 2 mg/mL GCBD NPs were mixed with 500 µCi 125I, and then the mixture was mixed with blow-dried Idongen for 20 min with shaking. After the reaction, the mixture was washed three times with 100 kDa ultra-filtration tube.

#### Radiolabeling stability

The radiolabeling stability of ^125^I labeled GCBD NPs was then evaluated by incubating ^125^I @GCBD NPs in serum or PBS for 48 h. The unlabeled radionuclides were washed with 100 kDa ultra-filtration tube for several times. The residual nuclide dose in the solution at different time points was detected by gamma counter (PerkinElmer) for radiostability.

### Detection of hydroxyl radical and GSH

#### Generation of ·OH catalysed by GCBD NPs

GCBD NPs (100 µg/mL for GCB NDs) were incubated with disodium terephthalate (TA) and H_2_O_2_ on a shaking table at 350 rpm and 37 °C for 3 h. The final concentrations of TA and H_2_O_2_ in the reaction system were 1.5 mM and 2 mM, respectively. TA + H_2_O_2_, TA + GCBD NPs, and H_2_O_2_ + GCBD NPs were used as controls. The fluorescence emission peaks at 435 nm (λex = 315 nm) were detected by fluorescence spectra of all samples.

#### Depletion of intracellular GSH

To detect the depletion of GSH by GCB NDs and GCBD NPs at the cellular level, 4T1 cells were cultured in 6-well plates for 24 h. Then, cells were cocultured with GCB NDs (n = 3) and GCBD NPs (n = 3). The blank group without nanomaterials was used as a control, respectively. After 24 h, cells were collected by centrifugation at 1000 rpm. The GSH content in each group was detected according to the instructions of the GSH/GSSG assay kit (Beyotime Biotechnology).

### Cellular experiments

#### Cytotoxicity

CCK-8 assay was used to detect the biosafety and cytotoxicity of NPs. Generally, 4T1 cells and human umbilical vein epithelial cells (HUVECs), which were cultured in DMEM/high glucose medium (10% fetal bovine serum and 1% penicillin–streptomycin), were cultivated in 96-well plates and then put in a cell incubator (37 °C, 5% CO_2_) for 24 h. For biosafety evaluation, GCB NDs with different concentration gradients were added to the plates with HUVECs. For cytotoxicity evaluation, GCB NDs, DOX, GCBD NPs with different concentration gradients and blanks were cocultured with 4T1 cells. After 24 h of culture, the relative cell viability was tested by CCK-8 assay.

#### Detection of cellular ·OH

4T1 cells were treated with DCFH-DA (10 µM, dispersed in DMEM medium) for 20 min. After washing with PBS for three times, samples were then separated into three groups: blank group cultured only with DMEM medium, experimental group incubated with GCB NDs (20 µg/mL) and GCBD NPs (6 µg/mL for DOX) for 3 h. Then, all samples were imaged by fluorescence microscope (OLYMPUS IX73).

#### Detection of H_2_O_2_

The concentration of H_2_O_2_ in cells were tested according to the instruction of hydrogen peroxide assay kit from Beyotime Biotechnology. Specifically, 4T1 cells were pre-cultivated in 6-well plates and then respectively incubated with DOX, GCB NDs or GCBD NDs ([DOX] = 2.5 µg/mL, [GCB] = 25 µg/mL) for 12 h (n = 3). The cells in each well were respectively washed by PBS for two times, collected by pancreatin into tubes, centrifuged and then added with 200 µL lysate for homogenate. The cellular homogenate was centrifuged at 12,000*g* to collect the supernatant. Afterwards, 100 µL of each supernatant and 100 µL hydrogen peroxide detection reagent was added to each well in the 96-well plate. After 30 min, the absorbance value at 560 nm was tested by ELIASA.

#### Dead and live cell staining

In order to display the cytotoxicity of the materials more intuitively, live and dead staining was used to further verify apoptosis. 4T1 cells were cocultured with DOX, GCB NDs or GCBD NPs ([DOX] = 2.5 µg/mL, [GCB] = 100 µg/mL) for 24 h, respectively. Afterwards, cells in different groups were stained with Calcein-AM (2 µM) and PI (4.5 µM) for 15 min and then observed by fluorescence microscope (OLYMPUS IX73).

#### Cellular uptake of DOX

4T1 cells were incubated with free DOX or GCBD NPs ([DOX] = 2 µg/mL) in 24-well plates with DMEM/high glucose medium. After incubation at different time points for 1, 6, 12, and 24 h, the medium was removed. Then, the 4T1 cells were washed with PBS for 3 times and fixed with 4% paraformaldehyde for 15 min. Then, the nuclei were stained with 4′,6-diamidino-2-phenylindole (DAPI) for 15 min. After that, the cells were washed with PBS for 3 times and finally photographed with confocal laser microscope (FV1200).

#### Cell apoptosis

4T1 cells were first incubated in 6-well plates for 24 h. Then, cells were incubated with GCB NDs or GCBD NPs for 24 h, and the group without materials was used as the control. Cells were collected by centrifugation at 1000 rpm and then stained with Annexin V-FITC and PI at 20–25 °C for 20 min. Finally, the apoptosis of 4T1 cells was detected by flow cytometry (BD FACSVERSE).

### Blood circulation and biodistribution

#### Blood circulation of GCBD NPs

GCBD NPs labeled with ^125^I (^125^I@GCBD NPs) were injected into tumor-bearing BALB/c mice (n = 3) through the tail vein for blood circulation and tissue distribution, respectively. For blood circulation, 10 µL of blood was collected from the orbit of mice, and the blood weight was recorded. The radiation intensity of blood was measured by gamma counter (PerkinElmer).

#### Biodistribution of GCBD NPs

GCBD NPs were injected into BALB-c mice (n = 3) through the tail vein to study the distribution of the material in vivo by measuring the intensity of radioactivity of ^125^I. The radiation intensity in the heart, liver, spleen, lung, kidney and tumor of the sacrificed mice was measured by gamma counter (PerkinElmer) after the injection of ^125^I@GCBD NPs for 4 and 24 h, respectively.

#### In vivo imaging

In order to reflect the enrichment of materials in mice more intuitively, GCB NDs and GCBD NPs were labeled with Cy5.5 and then respectively injected into tumor-bearing mice through the tail vein. Then, the mice with each treatment were imaged by IVIS (PerkinEimer) at 0 h, 1 h, 4 h, 12 and 24 h. After injection for 24 h, all mice were sacrificed to collect their hearts, livers, spleens, lungs, kidneys and tumors.

### Combined chemo/chemodynamic therapy of tumor

In order to verify the effect of tumor therapy in vivo, 4T1 cells were injected into the back of BALB-c mice to establish 4T1 tumor-bearing mouse models. When the tumor volume reached approximately 50–100 mm^3^, the mice were randomly divided into four groups (n = 6): (1) PBS, (2) DOX (5 mg kg^–1^), (3) GCB NDs (18.72 mg kg^–1^), and (4) GCBD NPs (5 mg kg^–1^ of DOX) and respectively injected with specific solutions through the tail vein. On day 7 post treatment, one mouse in each group was randomly sacrificed, and the tumor was subjected to TUNEL staining to test the therapeutic effect of the drugs. One mouse in each group was randomly sacrificed for H&E-stained slices of its heart, liver, spleen, lung, kidney and tumor after the treatment to test whether drugs have obvious toxic and side effects on normal tissues and 4T1 tumors of mice. The tumor volume and the weight of the mice were recorded every other day for 14 days. All the mice were sacrificed after the treatment, and the 4T1 tumors were removed, weighed and photographed.

## Results and discussion

### Synthesis and characterization of BSA nanocarriers

**Scheme 1:**
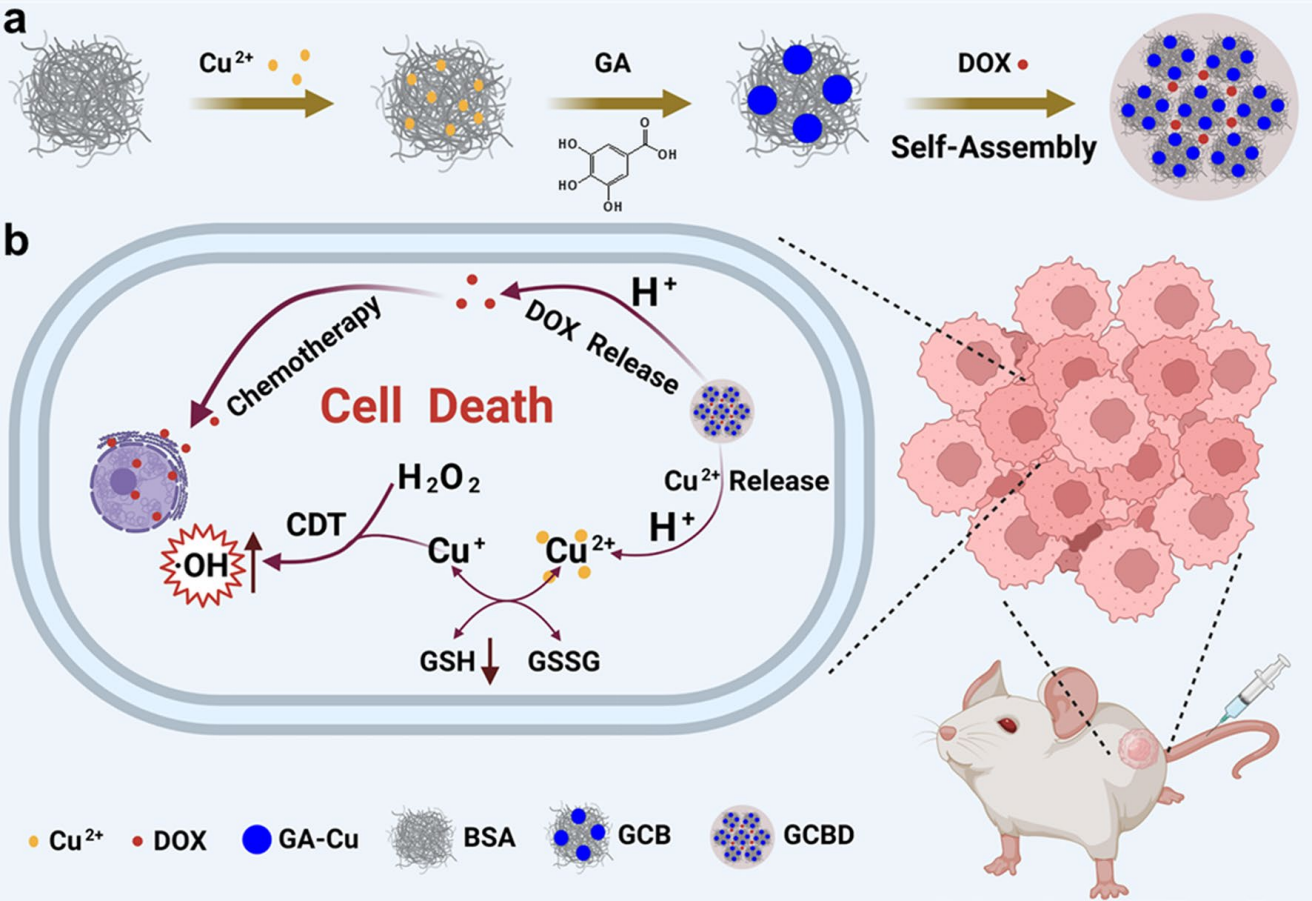
Schematic illustration of **a** the synthesis procedure of GA-Cu@BSA-DOX nanoparticles (GCBD NPs) and **b** the tumor microenvironment (TME)-responsive chemo/chemodynamic cancer therapy

**Fig. 1:**
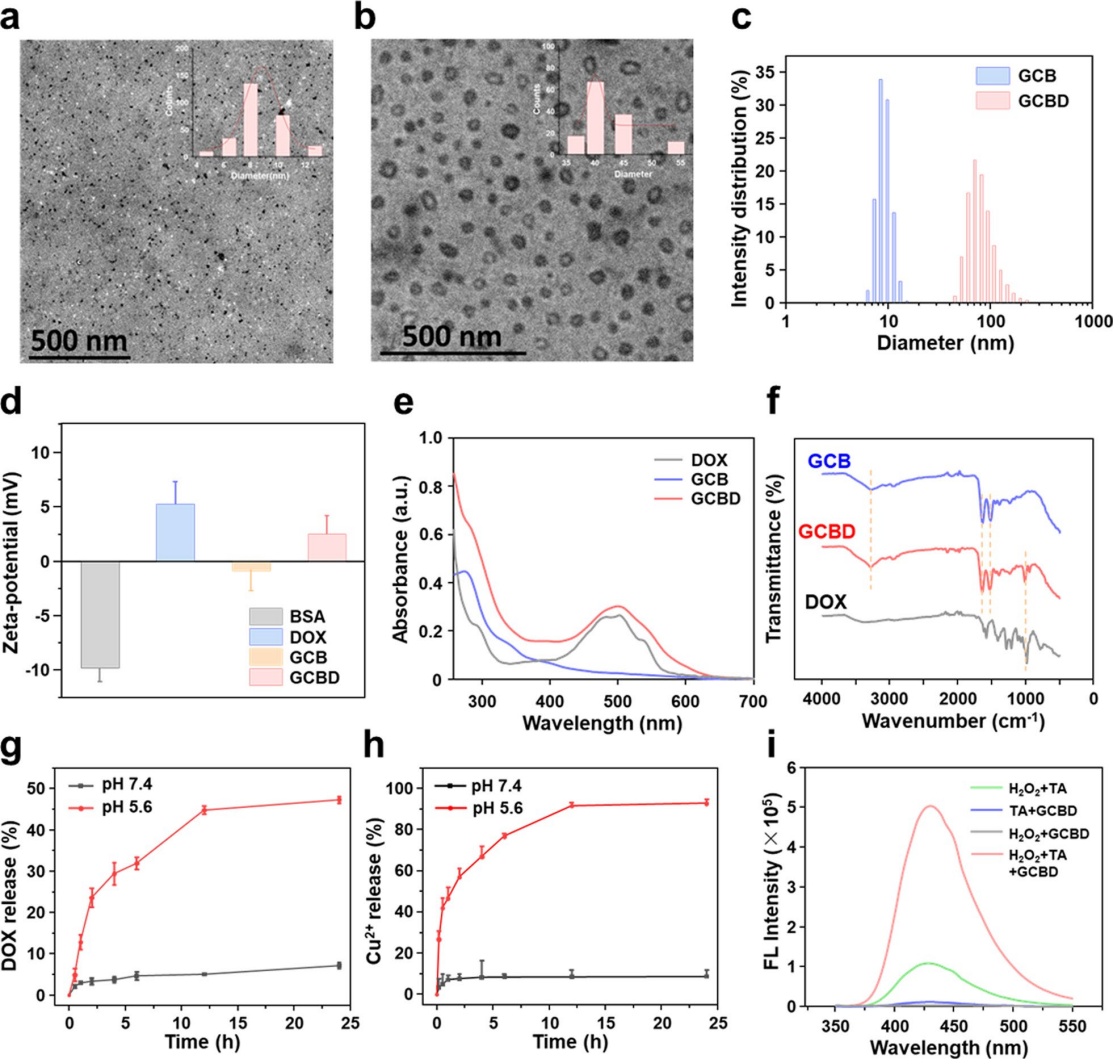
Characterization of GA-Cu@BSA (GCB NDs) and GA-Cu@BSA-DOX (GCBD NPs). **a** Transmission electron microscope (TEM) image of GCB NDs showing an average size of 8.1 nm. **b** TEM image of GCBD NPs showing an average size of 40.1 nm. **c** Dynamic light scattering (DLS) of GCB NDs and GCBD NPs. **d** The zeta potential of free BSA, free DOX, GCB NDs and GCBD NPs. **e** The absorbance spectra of free DOX, GCB NDs and GCBD NPs. **f** Fourier transform infrared (FT-IR) spectra of free DOX, GCB NDs and GCBD NPs. **g** The release profile of DOX from GCBD NPs at different pH. **h** The release profile of copper ions from GCBD NPs at different pH. **i** The fluorescence spectra of different solutions including TA + H_2_O_2_, TA + GCBD NPs, H_2_O_2_ + GCBD NPs and H_2_O_2_ + TA + GCBD NPs (λex = 315 nm)

The synthesis procedure of GA-Cu@BSA-DOX nanoparticles (GCBD NPs) for TME-responsive chemo/chemodynamic cancer therapy is illustrated in Scheme 1. Briefly, copper ions were attached onto bovine serum albumin (BSA) through electrostatic interactions, which were then solidified by gallic acid (GA) chelation in situ to form GA-Cu@BSA nanodots (GCB NDs). Transmission electron microscope (TEM) imaging showed that GCB NDs were evenly distributed with an average size of 8.1 nm, which was larger than that of PVP-stabilized GA-Cu nanodots (GC NDs), indicating that BSA functioned as the template rather than surfactant during the reaction (Fig. 1a, Additional file 1: Fig. S1). The chemical groups in GCB NDs were analyzed by Fourier transform infrared (FT-IR) spectrometry (Additional file 1: Fig. S2). The characteristic peaks of GCB NDs were similar to those of BSA, indicating the presence of BSA in the GCB NDs. Meanwhile, the characteristic peaks of 3290 cm^–1^ (O–H stretching) and 1030 cm^–1^ (O–H of the carboxylic group) for GA vanished in the FT-IR spectra of GCB NDs, revealing that the copper ions were chelated with the phenolic hydroxyl and carboxyl groups of GA.

### DOX loading onto GCB NDs

GCB NDs were then evaluated as BSA nanocarriers to load FDA-approved chemodrug (DOX) at different mass ratios, achieving GCBD NPs through hydrophobic interactions by stirring overnight in the dark. The DOX loading efficiency gradually increased as the mass ratio of DOX to GCB NDs increased until a plateau of approximately 30.12% for GCBD NPs (Additional file 1: Fig. S3). TEM images of GCBD NPs showed that DOX encapsulation resulted in an enlarged average size of 40.1 nm (Fig. 1b). Dynamic light scattering (DLS) determined that the average hydrodynamic size increased from 10 nm for GCB NDs to 60 nm for GCBD NPs (Fig. 1c), which was consistent with the TEM results, while the slightly larger hydrodynamic size was reasonable due to the hydration of nanomaterials in solution. Since DOX was positively charged, the negatively charged GCB NDs turned into positively charged GCBD NPs after DOX loading (Fig. 1d). In addition, the absorbance spectra of GCBD NPs demonstrated the characteristic absorbance peaks of BSA at 275 nm, and DOX redshifted to about 500 nm, preliminarily confirming that DOX was loaded onto the BSA nanocarriers (Fig. 1e). Furthermore, in contrast to GCB NDs, the FT-IR spectra of GCBD NPs showed the characteristic peaks of DOX at 3421 cm^–1^ (O–H stretching vibration peak), 1652 cm^–1^ (C–C ring tension stretching vibration peak), 1618 cm^–1^ (C=O stretching vibration peak), 1406 cm^–1^ (C–H bending vibration peak), 1284 cm^–1^ (C=N stretching vibration peak), 1211 cm^–1^ (C–O–C stretching vibration peak) and 987 cm^–1^ (N–H stretching vibration peak), validating the successful DOX loading onto the BSA nanocarriers (Fig. 1f).

To evaluate the elemental composition and valence state of the as-prepared nanomaterials, X-ray photoelectron spectroscopy (XPS) was conducted after complete purification (Additional file 1: Fig. S4). All GC NDs, GCB NDs and GCBD NPs showed the characteristic peaks of C 1s (284.8 eV), N 1s (399.8 eV), O 1s (531.7 eV), and Cu 2p (934.4 eV), indicating the successful chelation and solidication of copper ions by GA. The higher ratio of N in GCB NDs than in GC NDs was due to the higher N content in BSA than in PVP, while the higher ratio of C and O in GCBD NPs than in GCB NDs stemmed from the encapsulation of DOX. To further determine the valence state of copper, the corresponding high-resolution Cu 2p spectra were obtained and analyzed. The two dominant peaks at 934.3 and 954.2 eV could be ascribed to Cu 2p3/2 and Cu 2p1/2 of Cu^2+^, while the satellite peaks in the range of 938–948 eV were testified to be the typical peaks of Cu^2+^ (Additional file 1: Fig. S5). The copper content in the GCBD NPs was 0.94 ± 0.24 wt% as determined by inductively coupled plasma–optical emission spectrometry (ICP–OES), which was in accordance with that semiquantified from the XPS results.

### pH-sensitive release of therapeutic agents and production of hydroxyl radicals

The promise of GCBD NPs for TME-triggered therapy was then evaluated. Considering the relatively acidic environment in tumors (pH = 5.6), we investigated the release profile of copper ions and DOX from GCBD NPs at different pH values. The time-dependent release of DOX from GCBD NPs was evaluated by testing the absorbance spectra of solutions outside the dialysis bags (Fig. 1g). At pH 7.4, less than 7.2% of DOX was released from GCBD NPs after incubation for 24 h. Meanwhile, at pH 5.6, the DOX release profile of GCBD NPs showed a sharp increase in the first 2 h and achieved as high as 47.3% after 24 h, indicating the promise of GCBD NPs for TME-responsive chemotherapy.

The time-dependent release of copper ions from GCBD NPs was evaluated by copper determination kit (Fig. 1h). GCBD NPs could release about 92.7% of copper ions under acidic conditions and less than 9% under neutral conditions after 24 h. The significantly greater release of copper ions under acidic conditions is promising for the TME-sensitive release of sufficient copper ions, which could catalyze Fenton-like reactions to produce cytotoxic ·OH for chemodynamic therapy (CDT). Subsequently, the concentration of ·OH produced from H_2_O_2_ at a tumoral concentration (1 mM) was investigated by disodium terephthalate (TA), which could react with ·OH to produce TAOH with a fluorescence peak at 435 nm (λex = 315 nm) (Fig. 1i). Although a slight signal of TAOH appeared in free H_2_O_2_, a much stronger fluorescence intensity emerged after incubating GCBD NPs with H_2_O_2_ for 3 h, suggesting the capability of GCBD NPs for specific CDT of cancer cells.

Furthermore, the physiological stability of the as-prepared nanomaterials was evaluated. GC NDs, GCB NDs and GCBD NPs were lyophilized and redispersed in water, resulting in clear solutions with specific colours (Additional file 1: Fig. S6), which is promising for clinical translation. The GCB NDs and GCBD NPs in different solutions (water, PBS and DMEM medium) were monitored for 7 days, showing steady hydrodynamic diameter and PDI with negligible agglomeration or precipitation (Additional file 1: Fig. S7). These results all together supported the further evaluation of BSA-based nanomaterials for biomedical applications.

### Chemo/chemodynamic therapy of cancer cells

**Fig. 2:**
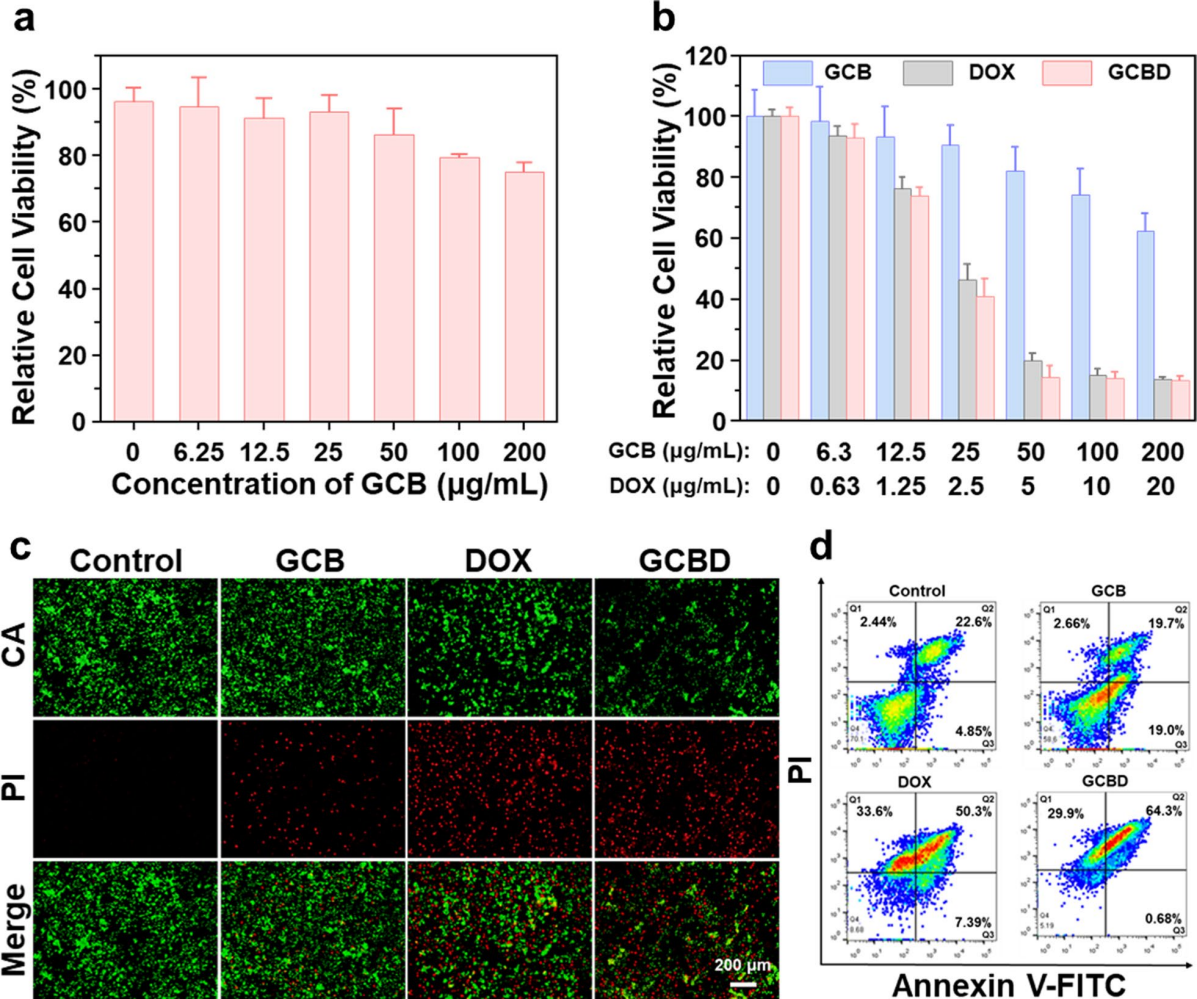
The analysis of cell death pathway. **a** Relative cell viability of HUVEC cells after incubation with GCB NDs at different concentrations (0–200 µg/mL) for 24 h. **b** Relative cell viability of 4T1 cells after incubation with GCB NDs (0–200 µg/mL), DOX (0–20 µg/mL) or GCBD NPs (0–20 µg/mL of DOX) for 24 h. **c** Confocal imaging of calcein-AM/PI stained 4T1 cells after incubation with different solutions for 24 h. **d** The flow cytometry analysis of 4T1 cells after incubation with different solutions for 24 h, showing more significant apoptosis in GCBD NPs-treated cells than other groups (2.5 µg/mL for DOX)

We then evaluated the promise of GCBD NPs for combined chemo/chemodynamic therapy in vitro. Before conducting chemo/chemodynamic therapy of cancer cells, we first assessed the biocompatibility of the BSA nanocarriers to normal cells. After incubation with GCB nanodots for 24 h, the relative cell viability of human umbilical vein epithelial cells (HUVECs) was over 75%, even at the highest concentration of 200 µg/mL (Fig. 2a), encouraging us to further study the therapeutic efficacy of these nanomaterials for cancer cells. For mouse breast cancer cells (4T1 cells), GCB nanodots showed limited toxicity, leaving about 62% cells alive at a concentration of 200 µg/mL (Fig. 2b). Meanwhile, GCBD NPs showed a more significant lethal effect on cancer cells than GCB nanodots, resulting in less than 20% relative viability of 4T1 cells at the highest concentration ([DOX] = 20 µg/mL, [GCB] = 200 µg/mL) (Fig. 2b). Confocal imaging of CA/PI-stained 4T1 cells also revealed maximum cancer cell death after incubation with GCBD NPs, indicating the potent efficacy of combined chemo- and chemodynamic therapy of cancer cells (Fig. 2c and Additional file 1: Fig. S8a). Flow cytometry was then conducted to analyze the cell death pathway, showing that the apoptosis rate of cells after GCBD NP treatment was significantly higher than those treated with PBS or GCB NDs and even higher than those treated with free DOX (Fig. 2d, and Additional file 1: Fig. S8b), which was consistent with the cytotoxicity and confocal results.

**Fig. 3:**
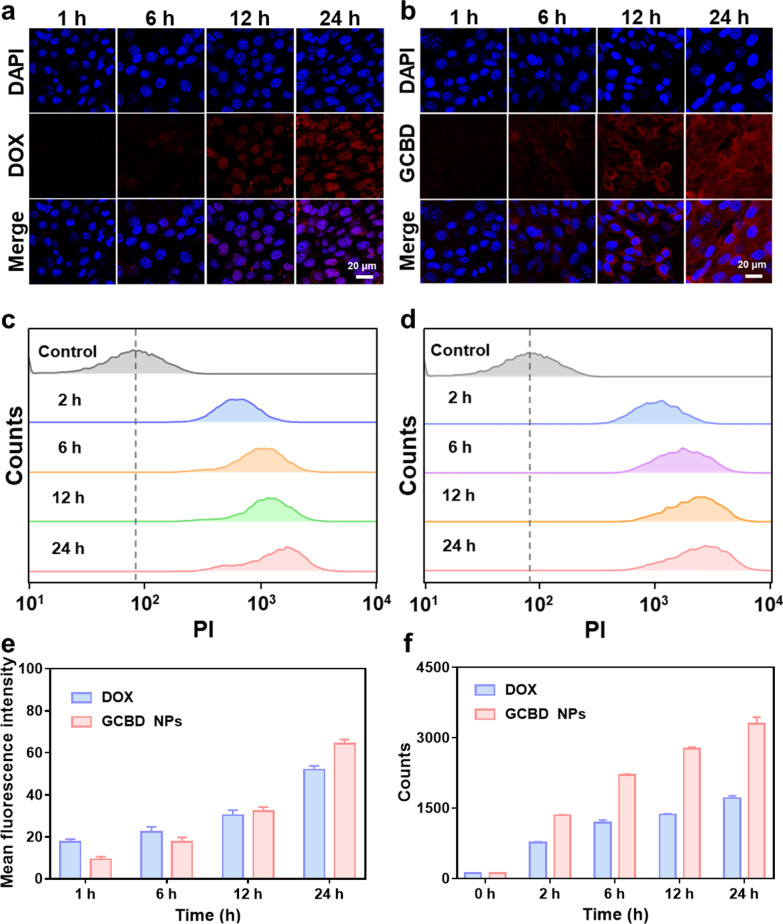
Cellular uptake of chemotherapy drug. **a**, **b** Confocal imaging of 4T1 cells demonstrating the cellular uptake of DOX (2 µg/mL) (**a**) and GCBD NPs (2 µg/mL of DOX) (**b**) at different time points. The nuclei of cells were stained with DAPI. **c**, **d** Flow cytometry-measured cellular uptake of free DOX (2 µg/mL) (**c**) and GCBD NPs (2 µg/mL of DOX) (**d**) in 4T1 cells at different time points. **e** The semiquantitative analysis of cellular uptake corresponding to the confocal images in **a**, **b**. **f** The semiquantitative analysis of cellular uptake corresponding to the flow cytometry results in **c**, **d**

To trace the mechanism for the stronger cytotoxicity of GCBD NPs to cancer cells, we first evaluated the cellular uptake of DOX by confocal imaging and flow cytometry. Confocal imaging showed that the fluorescence of free DOX was brighter with longer incubation time, especially in the cell nuclei (Fig. 3a). Meanwhile, for GCBD NP-treated cells, DOX fluorescence quickly appeared in the lysosome after 4 h and ultimately in the nuclei at 24 h of incubation (Fig. 3b), suggesting the release of DOX in acidic cellular lysosomes for entering nuclei to bind with DNA. Flow cytometry (Fig. 3c, d), consistent with the semiquantitative analysis (Fig. 3e, f), showed stronger DOX fluorescence intensity in GCBD NP-treated cells than in cells treated with free DOX at each time point. These results indicated more cellular uptake of DOX with the delivery of BSA nanocarriers, which is beneficial for enhancing chemotherapy efficacy.

**Fig. 4:**
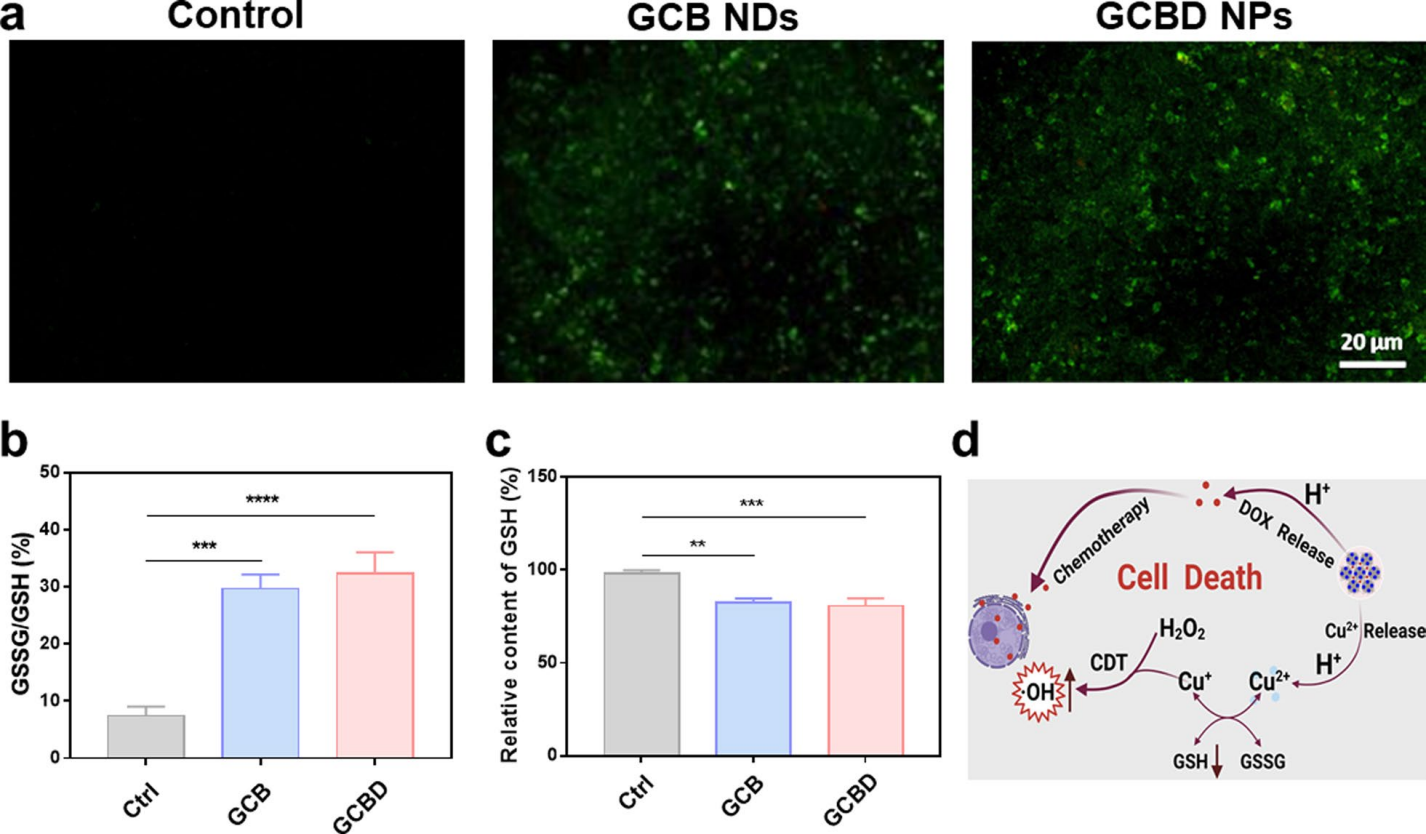
In vitro chemo/chemodynamic therapy based on GCBD NPs. **a** Confocal laser scanning microscopy (CLSM) imaging of 4T1 cells stained with DCFH-DA after various treatments. Scale bar: 20 μm. **b** The GSSG/GSH ratio of GSH solutions (10 mM) after incubation with PBS, GCB NDs or GCBD NPs for 24 h. **c** The relative GSH content in 4T1 cells after incubation with different solutions for 24 h. **d** Schematic illustration showing the mechanism for cellular chemo/chemodynamic therapy based on GCBD NPs. P values in (**b**, **c**) were calculated by multiple t tests (****P < 0.0001, ***P < 0.001, ***P < 0.001, **P < 0.01, *P < 0.05)

Considering the capability of GCBD NPs to catalyze the decomposition of H_2_O_2_ into ·OH for chemodynamic therapy, DCFH-DA was then applied as the fluorescent probe to detect the ROS level in cancer cells. The confocal imaging illustrated obvious green fluorescence in 4T1 cells after incubation with GCB NDs, while those treated with GCBD NPs demonstrated even more significant ROS signal (Fig. 4a). The stronger ROS production by GCBD NPs than GCB NDs could be ascribed to DOX, which could activate cellular NOXs to generate oxygen radical and subsequent more hydrogen peroxide to further fuel CDT (Additional file 1: Fig. S9). Since the presence of GSH would compromise the toxicity of ·OH to cells, we then examined the cellular concentration of GSH by a GSH/GSSG detection kit. Compared with the blank group, GCB NDs and GCBD NPs with copper ions could effectively consume GSH (Fig. 4b), resulting in chemically inert GSSG (Fig. 4c). These results verified that GCBD NPs could catalyze significant production of cytotoxic ROS and convert the high expression of GSH into GSSH, suggesting great promise for TME-responsive chemo/chemodynamic therapy (Fig. 4d).

### Blood circulation and biodistribution of GCBD NPs in mice

**Fig. 5:**
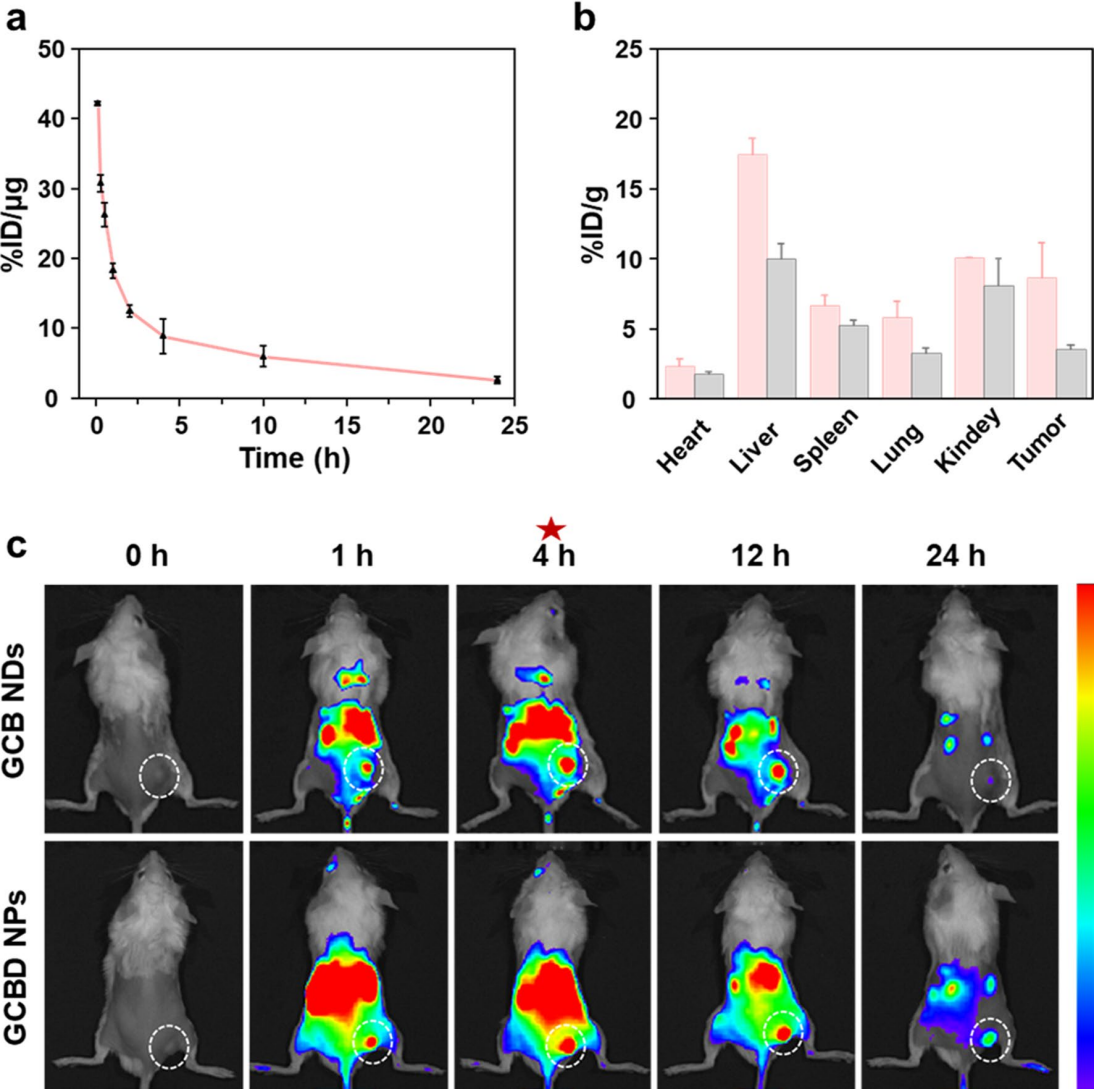
The blood circulation and biodistribution of GCBD NPs in mice. **a** The blood circulation of ^125^I@GCBD NPs in Balb-c mice after intravenous (i.v.) injection (20 µCi for ^125^I, 2.5 mg kg^−1^ of DOX). **b** The biodistribution of ^125^I-labeled GCBD NPs at 4 h (pink) and 24 h (gray) after i.v. injection (20  µCi for ^125^I, 2.5 mg kg^−1^ of DOX) as determined by measuring the radionuclide intensity in the major organs and tumors. **c** The fluorescence imaging of 4T1 tumor-bearing mice at different time points after i.v. administration of Cy5.5-labeled GCB NDs (10 mg kg^−1^) or GCBD NPs (2.5 mg kg^−1^ of DOX) (the tumor was highlighted by white dotted circle). The error bars were based on the standard deviation of tree mice in each group

To evaluate the blood circulation and biodistribution of GCBD NPs in mice, radionuclide ^125^I was labeled onto GCBD NPs, showing ideal labeling stability in both serum and PBS within 48 h (Additional file 1: Fig. S10). After intravenous administration into healthy Balb-c mice, GCBD NPs demonstrated a circulation half-life of 1.05 ± 0.18 h (Fig. 5a). Meanwhile, the ex vivo radionuclide intensity showed that the tumoral accumulation of GCBD NPs reached 8.7% at 4 h and 3.5% at 24 h post i.v. injection (Fig. 5b).

On the other hand, the fluorescence imaging of Cy5.5-labeled GCB NDs and GCBD NPs in tumor-bearing mice exhibited the highest tumoral accumulation at 4 h post administration (Fig. 5c). Besides, GCBD NPs showed longer retention time in tumors due to their larger size. Moreover, the fluorescence imaging of mice, consistent with the radionuclide intensity of major organs, demonstrated the gradually decreased signal of GCBD NPs in the liver, kidney and spleen over time, preliminarily indicating the rapid metabolism of GCBD NPs in mice. These results together promised GCBD NPs for combined therapy of tumors.

### Chemo/chemodynamic therapy of tumor

**Fig. 6:**
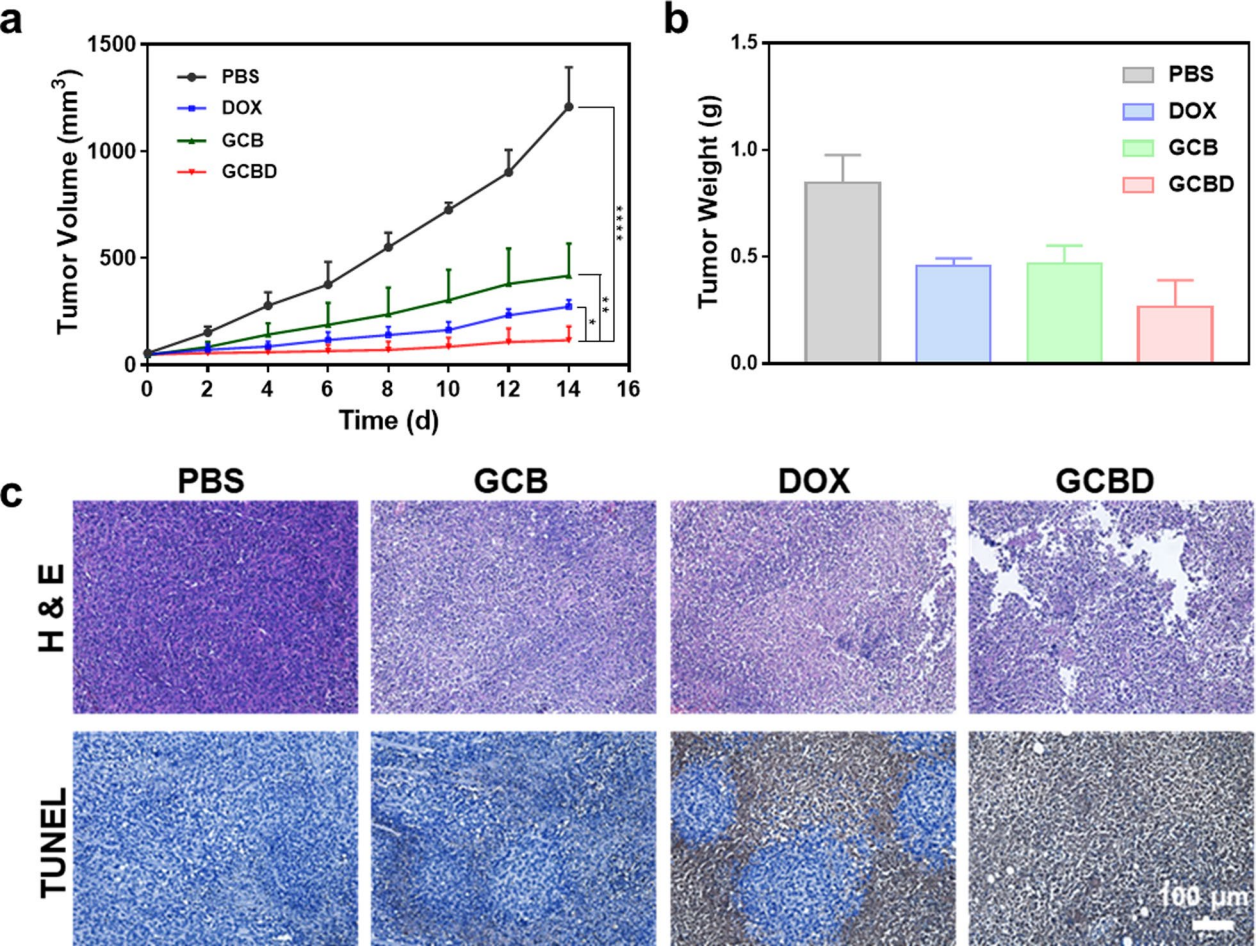
Combined chemo/chemodynamic therapy in vivo. **a** Tumor growth curves monitored for 14 days after various treatments. **b** Tumor weight in each group 14 days after each treatment. **c** Hematoxylin and eosin (H&E) and TUNEL stained tumor slice post specific treatment. The error bars were based on the standard deviation of 6 mice in each group. P values in (**a**) were calculated by multiple t tests (****P < 0.0001, ***P < 0.001, ***P < 0.001, **P < 0.01, *P < 0.05)

The 4T1 tumor-bearing mice were randomly divided into four groups (6 mice in each group) when the size of inoculated tumors reached 50 mm^3^, which were i.v. injected with PBS, free DOX, GCB NDs or GCBD NPs. The tumoral size of the mice was monitored by caliper over the next 14 days. Although free DOX or GCB could moderately restrain the tumor volume, GCBD NPs could inhibit the growth of tumors more effectively due to the combination of chemodrug and CDT agents (Fig. 6a). The tumors excised from mice after 14 days also demonstrated the best therapeutic efficacy by combining chemo/chemodynamic therapy based on GCBD NPs (Fig. 6b, Additional file 1: Fig. S11). Hematoxylin and eosin (H&E) and TUNEL stained tumor slices revealed the most obvious cellular necrosis based on GCBD NPs compared with other groups (Fig. 6c), further confirming the synergistic effect of combined chemo/chemodynamic therapy.

The body weight of mice slightly declined during the first 2 days after systematic administration of GCBD NPs but recovered to a level as high as that of the PBS group after 14 days, preliminarily suggesting the biocompatibility of GCBD NPs to mice (Additional file 1: Fig. S12). Furthermore, the mice were sacrificed at 14 days post-treatment, and major organs and tissues were collected and stained with H&E (Additional file 1: Fig. S13). The histopathological analysis showed negligible inflammation or injury in each organ, further indicating the biosafety of GCBD NPs in mice during the monitoring period.

## Conclusions

In summary, we synthesized BSA-based nanocarriers named GCBD NPs for combining chemotherapy and CDT therapy of cancer. The prepared GCBD NPs with regular morphology and size showed high physiological stability for at least 1 week. Under acidic conditions, GCBD NPs specifically released 90% of copper ions, which could convert GSH into GSSG at the cellular level and catalyze H_2_O_2_ into cytotoxic hydroxyl radicals for CDT. Meanwhile, the cellular uptake of DOX was enhanced by the delivery of GCBD NPs, which further entered cellular nuclei by acid-enhanced release to induce more potent cancer cell death. The chemo and chemodynamic therapy together induced the most apoptosis of 4T1 cells, as revealed by CCK-8, immunofluorescence imaging and flow cytometry. With the help of radionuclide ^125^I labeling, the pharmacokinetics of GCBD NPs were monitored, showing a blood circulation half-life of 1.05 h and efficient tumoral accumulation, especially at 4 h post i.v. injection. After systematic administration, GCBD NPs showed the most effective inhibition of 4T1 tumor growth as compared to other groups, whose necrosis was further validated by TUNEL and H&E-stained tumor slices. Furthermore, H&E-stained slices of the major organs obtained 14 days after treatments preliminarily suggested that GCBD NPs had no obvious toxicity or side effects on mice. Our results indicated that combined chemotherapy and CDT therapy based on biocompatible nanocarriers is a promising cancer treatment strategy to achieve potent therapeutic efficacy with negligible adverse effects, which could be further extended to various nanoplatforms incorporating other metal ions and drugs.

## Supplementary Information

**Additional file 1: Figure S1.** TEM image of PVP-stabilized GA-Cu nanodots (GC NDs) (a) and GA-Cu@BSA (GCB NDs) (b). **Figure S2.** Fourier transform infrared (FT-IR) spectra of free BSA, free gallic acid (GA) and GCB NDs. **Figure S3.** Loading capacity of DOX by GCB NDs at different ratio of DOX to GCB. **Figure S4.** XPS spectra of GC NDs (a), GCB NDs (b) and GCBD NPs (c). **Figure S5.** The corresponding high-resolution Cu 2p spectra of GC NDs (a), GCB NDs (b) and GCBD NPs (c). **Figure S6.** Photos of lyophilized GC NDs, GCB NDs and GCBD NPs and the corresponding solutions. **Figure S7.** (a) The physiological stability of GCB NDs and GCBD NPs in different solutions (water, PBS and DMEM medium). (b) Hydrodynamic diameter and the corresponding PDI value of GCBD NPs in PBS measured by dynamic laser scattering (DLS). **Figure S8.** (a) Fluorescence intensity of PI obtained by ImageJ processing and (b) apoptosis rate of cells in each group as determined by flow cytometry. P values were calculated by multiple t tests. **Figure S9.** Hydrogen peroxide assay kit tested H_2_O_2_ content in 4T1 cells after incubation with DOX, GCB or GCBD. **Figure S10.** Radiolabeling stability of 125I@GCBD NPs in PBS and 10% FBS during 48 h. **Figure S11.** Photographs of tumors in each group 14 days after each treatment. **Figure S12.** Body weight curves of mice in different groups. **Figure S13.** H&E-stained slices of major organs in 4T1 tumor-bearing mice at 14 days post each treatment.

## Data Availability

All data used to generate these results is available in the main text and additional information.
