# Supplementary material for: Tumor microenvironment-responsive BSA nanocarriers for combined chemo/chemodynamic cancer therapy
Source: J Nanobiotechnology. 2022 May 12;20:223. doi: 10.1186/s12951-022-01442-5 (PMC9097166; doi:10.1186/s12951-022-01442-5)
Supplement: Supplementary file 1 — Additional file 1: Figure S1. TEM image of PVP-stabilized GA-Cu nanodots (GC NDs) (a) and GA-Cu@BSA (GCB NDs) (b). Figure S2. Fourier transform infrared (FT-IR) spectra of free BSA, free gallic acid (GA) and GCB NDs. Figure S3. Loading capacity of DOX by GCB NDs at different ratio of DOX to GCB. Figure S4. XPS spectra of GC NDs (a), GCB NDs (b) and GCBD NPs (c). Figure S5. The corresponding high-resolution Cu 2p spectra of GC NDs (a), GCB NDs (b) and GCBD NPs (c). Figure S6. Photos of lyophilized GC NDs, GCB NDs and GCBD NPs and the corresponding solutions. Figure S7. (a) The physiological stability of GCB NDs and GCBD NPs in different solutions (water, PBS and DMEM medium). (b) Hydrodynamic diameter and the corresponding PDI value of GCBD NPs in PBS measured by dynamic laser scattering (DLS). Figure S8. (a) Fluorescence intensity of PI obtained by ImageJ processing and (b) apoptosis rate of cells in each group as determined by flow cytometry. P values were calculated by multiple t tests. Figure S9. Hydrogen peroxide assay kit tested H2O2 content in 4T1 cells after incubation with DOX, GCB or GCBD. Figure S10. Radiolabeling stability of 125I@GCBD NPs in PBS and 10% FBS during 48 h. Figure S11. Photographs of tumors in each group 14 days after each treatment. Figure S12. Body weight curves of mice in different groups. Figure S13. H&E-stained slices of major organs in 4T1 tumor-bearing mice at 14 days post each treatment. [file 12951_2022_1442_MOESM1_ESM.docx]

**Additional information**

**Tumor microenvironment-responsive BSA nanocarriers for** **combined chemo/chemodynamic cancer therapy**

Ruiyi Zhang^1^, Teng Liu^2^*, Wanzhen Li^1^, Zhiyuan Ma^1^, Pei Pei^2^, Weiwei Zhang^1^, Kai Yang^2^*, Yugui Tao^1^*

^1^ School of Biological and Food Engineering, Anhui Polytechnic University, Wuhu, Anhui 241000, China

^2^ State Key Laboratory of Radiation Medicine and Protection, School of Radiation Medicine and Protection & School for Radiological and Interdisciplinary Sciences (RAD-X), Collaborative Innovation Center of Radiation Medicine of Jiangsu Higher Education Institutions, Soochow University, Suzhou, Jiangsu 215123, China

Correspondence: tliu13@suda.edu.cn, kyang@suda.edu.cn and [swgctaoyg@126.com](mailto:swgctaoyg@126.com)


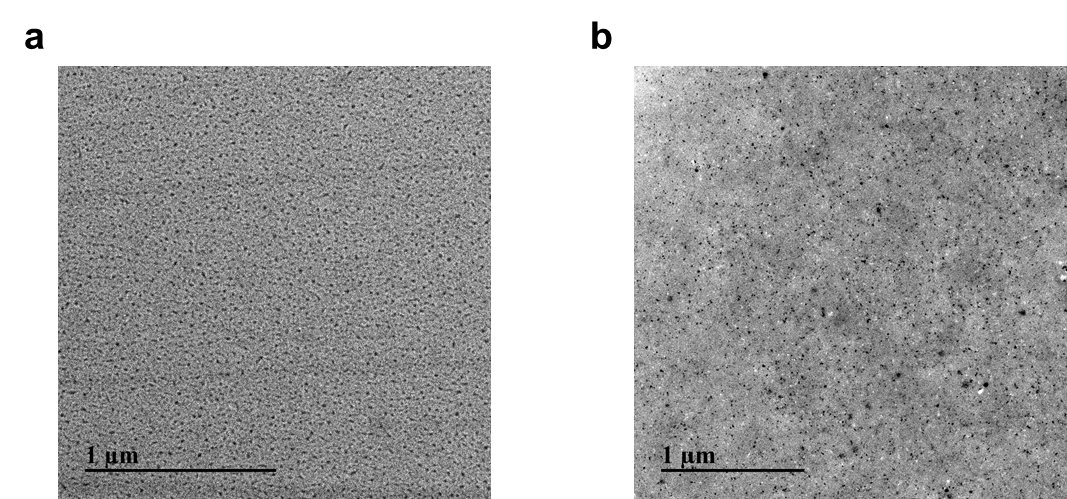


**Figure S1.** TEM image of PVP-stabilized GA-Cu nanodots (GC NDs) (a) and GA-Cu@BSA (GCB NDs) (b).


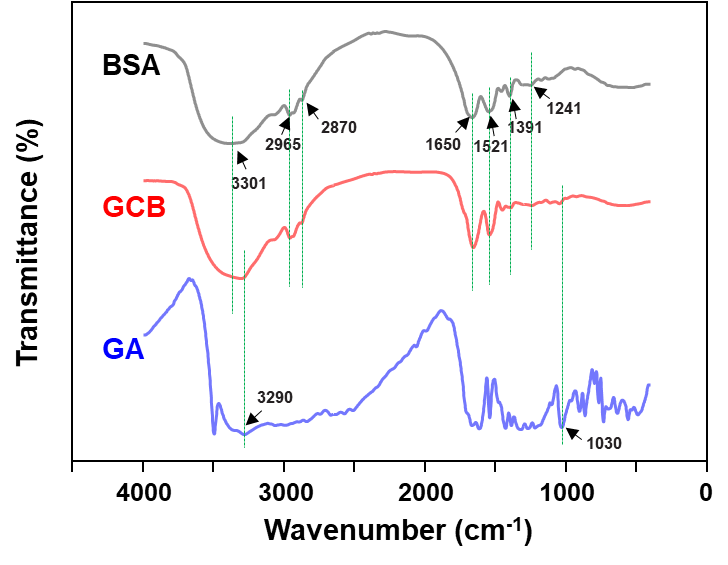


**Figure S2.** Fourier transform infrared (FT-IR) spectra of free BSA, free gallic acid (GA) and GCB NDs.


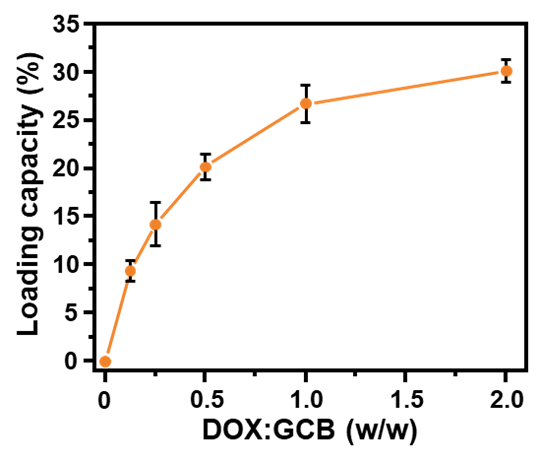


**Figure S3.** Loading capacity of DOX by GCB NDs at different ratio of DOX to GCB.


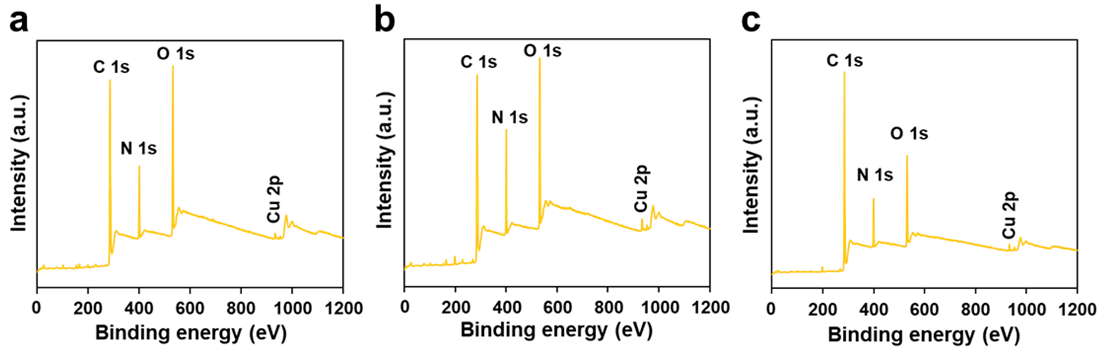


**Figure S4.** XPS spectra of GC NDs (a), GCB NDs (b) and GCBD NPs (c).


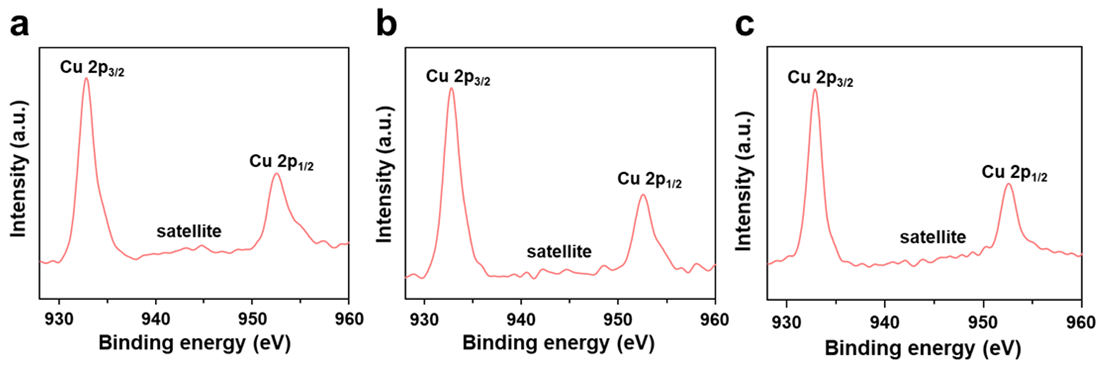


**Figure S5.** The corresponding high-resolution Cu 2p spectra of GC NDs (a), GCB NDs (b) and GCBD NPs (c).


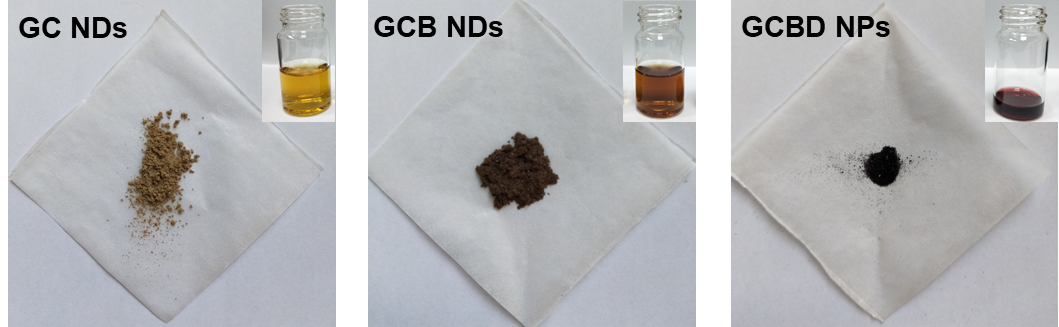


**Figure S6.** Photos of lyophilized GC NDs, GCB NDs and GCBD NPs and the corresponding solutions.


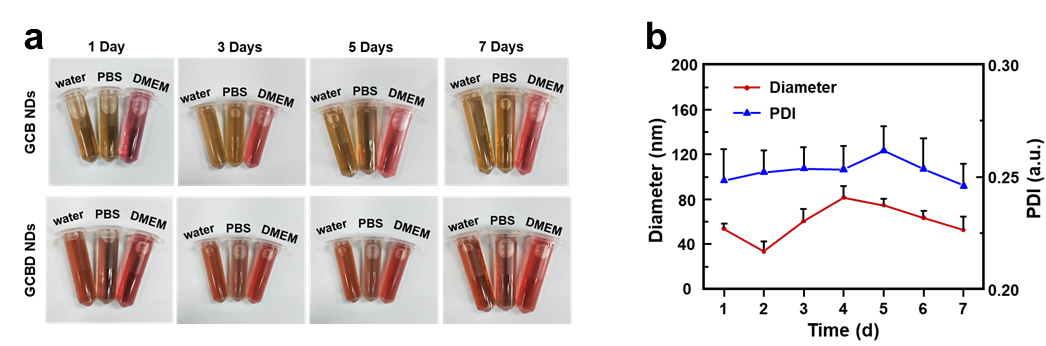


**Figure S7.** (a) The physiological stability of GCB NDs and GCBD NPs in different solutions (water, PBS and DMEM medium). (b) Hydrodynamic diameter and the corresponding PDI value of GCBD NPs in PBS measured by dynamic laser scattering (DLS).


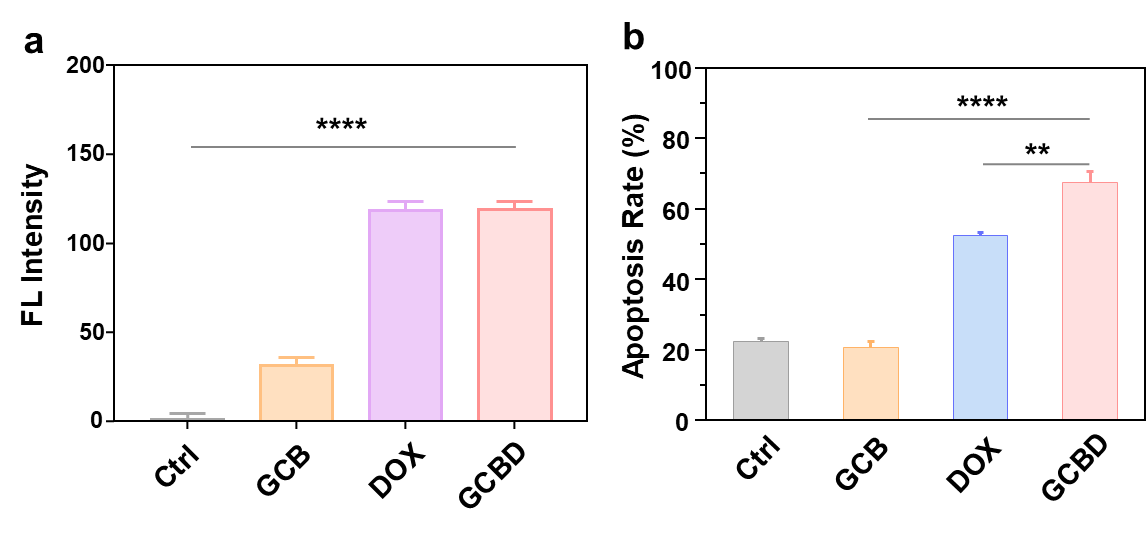


**Figure S8.** (a) Fluorescence intensity of PI obtained by ImageJ processing and (b) apoptosis rate of cells in each group as determined by flow cytometry. P values were calculated by multiple t tests.

**Figure S9.** Hydrogen peroxide assay kit tested H_2_O_2_ content in 4T1 cells after incubation with DOX, GCB or GCBD.


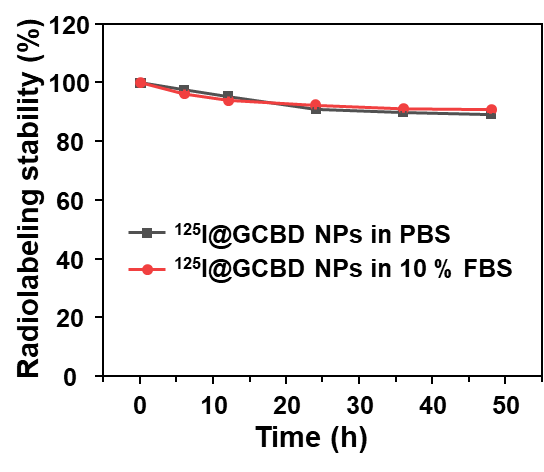


**Figure S10.** Radiolabeling stability of ^125^I@GCBD NPs in PBS and 10% FBS during 48 h.


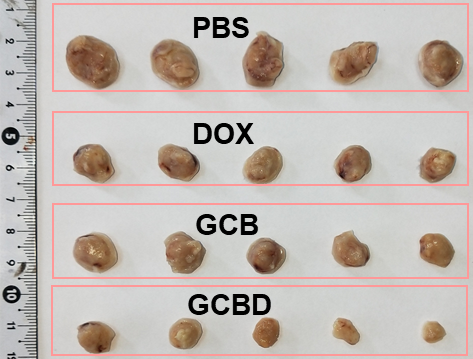


**Figure S11.** Photographs of tumors in each group 14 days after each treatment.

**Figure S12.** Body weight curves of mice in different groups.


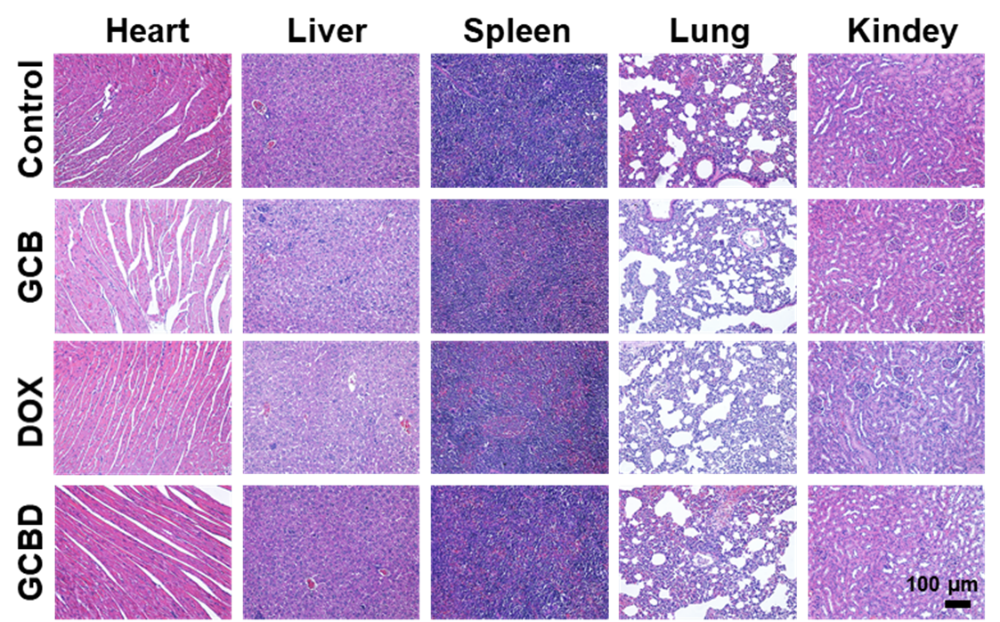


**Figure S13.** H&E-stained slices of major organs in 4T1 tumor-bearing mice at 14 days post each treatment.
